# The gut-eye axis in age-related macular degeneration: from microbial dysbiosis to targeted intervention strategies

**DOI:** 10.3389/ebm.2026.10876

**Published:** 2026-01-20

**Authors:** Nan Wang, Lin Luo, Xiaolan Yang

**Affiliations:** 1 Department of Ophthalmology, Qingdao Municipal Hospital, Qingdao Hospital of University of Health and Rehabilitation Sciences, Qingdao, China; 2 Department of Ophthalmology, The Second People’s Hospital of Jinan, Jinan, China

**Keywords:** age-related macular degeneration, fecalmicrobiota transplantation, gut microbiota, intestinal barrier, short-chain fatty acids

## Abstract

Age-related macular degeneration (AMD) represents a leading cause of irreversible blindness among the older persons. Characterized by a complex pathogenesis and multiple risk factors, AMD poses substantial challenges for treatment and has emerged as a significant public health concern. The gut microbiota constitutes a vast and dynamically evolving ecosystem, with a healthy microbial community playing an essential role in maintaining host homeostasis through its involvement in digestion and immune defense. However, alterations in microbial composition or function can compromise intestinal barrier integrity, trigger systemic inflammation, and contribute to disease pathogenesis. Evidence now underscores the influence of gut microbiota on the development and progression of AMD. This review examines the mechanisms by which gut microbes may contribute to AMD pathogenesis and evaluates the therapeutic potential of interventions targeting the gut microbiome—including dietary modifications, Pharmacological and Biological Agents, probiotics, prebiotics, and fecal microbiota transplantation—for AMD management.

## Impact statement

This manuscript aims to elucidate the role of the gut microbiome in the pathogenesis of age-related macular degeneration (AMD) through the “gut-eye axis” and to systematically position this emerging field. By synthesizing existing evidence, we comprehensively describe how gut dysbiosis drives the initiation and progression of AMD by compromising intestinal barrier integrity, triggering systemic inflammation, affecting the complement system, and altering microbial metabolite levels. Furthermore, this review evaluates the potential and challenges of gut microbiome-targeted therapeutic strategies, such as dietary modifications, prebiotics, probiotics, and fecal microbiota transplantation. The contribution of this work lies in providing a novel, interdisciplinary perspective for understanding and treating this complex ocular disease, linking gut health with retinal pathology and outlining future research directions for the field.

## Introduction

Age-related macular degeneration (AMD) is a progressive, multifactorial neurodegenerative disorder affecting the retina, predominantly manifesting in older adults. Epidemiological evidence identifies AMD as a leading cause of irreversible vision loss in aging populations across industrialized nations [1, 2]. With the global population aging, the prevalence of AMD has risen substantially, emerging as a critical public health challenge. Pathologically, AMD is categorized into two primary subtypes: dry (non-exudative) and wet (exudative) AMD. Dry AMD, the more common form, is defined by progressive geographic atrophy (GA) of the retinal pigment epithelium (RPE) and subsequent photoreceptor cell degeneration. When these pathological changes affect the macula, the central retinal region responsible for high-acuity vision, irreversible central vision loss ensues [3]. Wet AMD is characterized by the pathological invasion of choroidal capillaries through Bruch’s membrane into either the sub-RPE space or the neural retinal layer, culminating in choroidal neovascularization (CNV). These aberrant vessels exhibit heightened fragility and permeability, frequently leading to fluid leakage, retinal structural distortion, fibrotic scarring, and irreversible damage to the macular region. Collectively, these pathological changes drive progressive and often severe central vision loss [4]. Table 1 reports the comparison of the key characteristics between Dry and Wet AMD. AMD exhibits marked geographic variation in prevalence. Globally, among individuals aged ≥60 years, Dry AMD affects 8.5% of the population, while Wet AMD accounts for 2%. Epidemiological patterns demonstrate higher AMD rates in Europe and North America compared to Asia and Africa. Projections indicate the global AMD burden will surpass 350 million cases by 2040, reflecting demographic aging trends [5].

**TABLE 1:** Comparison of key characteristics between dry and wet AMD.

Feature	Dry (atrophic) AMD	Wet (neovascular) AMD
Prevalence	80%–90%	10%–20%
Disease onset & course	Insidious onset and slow progression	Acute onset and rapid progression
Core pathogenesis	Degeneration and atrophy of the retinal pigment epithelium (RPE)	Formation of a choroidal neovascularization (CNV) membrane beneath the RPE
Primary symptoms	Gradual, painless bilateral vision loss; may include metamorphopsia	Sudden vision loss, metamorphopsia, or central scotoma
Characteristic fundus findings	Drusen, pigmentary disturbances, geographic atrophy	Macular exudation, hemorrhage, subretinal/pigment epithelial fluid, disciform scar
Drusen characteristics	Hard: Small, round, well-defined borders Soft: Larger, poorly defined borders, may coalesce	Drusen may be present within or at the edge of the lesion
Late-stage features	Geographic atrophy of the RPE and choriocapillaris, unmasking of larger choroidal vessels	Organization of submacular hemorrhage leading to a disciform scar and permanent central vision loss
Complications	—	May be associated with macular edema; significant hemorrhage can lead to vitreous hemorrhage

AMD is a multifactorial condition associated with age, genetic predisposition, light exposure, immune status, sex, ethnicity, body weight, and oxidative stress. Nevertheless, its precise etiology remains incompletely understood. Currently, no effective treatment exists for the progressive degeneration and atrophy of photoreceptors and RPE cells in Dry AMD, which is managed primarily through nutritional supplementation and lifestyle modifications to delay disease progression [6]. In contrast, Wet AMD can be effectively controlled with anti-vascular endothelial growth factor (VEGF) agents such as bevacizumab, aflibercept, and ranibizumab. However, despite their efficacy in most cases, long-term anti-VEGF therapy poses a substantial socioeconomic burden [7, 8]. Moreover, approximately 10% of patients exhibit a poor response to anti-VEGF treatment, underscoring the need for novel therapeutic strategies [9].

The gut microbiota plays a crucial role in maintaining host physiological homeostasis by participating in nutrient metabolism and supporting innate immunity [10–12]. It contributes to a dynamic host-microbe equilibrium, wherein microbe-associated molecular patterns (MAMPs) can potentiate inflammatory responses [13]. Furthermore, gut-derived immune cells or damage-associated molecular patterns (DAMPs) may amplify the cascade of ocular inflammation [14, 15].

Disruption of the gut microbiota has been significantly associated with ocular diseases, including AMD [16, 17]. AMD is characterized by RPE dysfunction and photoreceptor loss, and its development can be influenced by various factors, among which diet plays a critical role [18]. Both animal and clinical studies have established a link between the gut microbiota and neovascular AMD. Dietary habits modulate the composition of the gut microbiota, which may in turn affect the progression of AMD [19–21]. High-glycemic index diets represent a significant risk factor for the development and progression of non-diabetic individuals. In animal studies, this type of diet has been associated with specific alterations, including reduced pigmentation and loss of RPE, accumulation of lipofuscin, and degeneration of photoreceptor cells [22, 23]. Studies have also revealed an increase in intestinal pro-inflammatory bacteria, such as *Anaerorhabdus* and *Oscillibacter*, which contributes to enhanced intestinal permeability [24, 25]. Furthermore, a reduction in glutamate, the primary excitatory neurotransmitter in the retina, is associated with impaired retinal neurotransmission, while elevated arginine levels are linked to progressive chorioretinal atrophy [26, 27]. Collectively, these findings demonstrate a significant association between alterations in the gut microbiota and the pathogenesis of AMD.

The Gut-Eye axis operates within a complex, multi-layered context. Several established risk factors for AMD, such as smoking and poor nutrition, particularly diets high in processed foods, sugars, and saturated fats—also act as potent disruptors of intestinal microbial homeostasis. Smoking induces gut dysbiosis, reduces microbial diversity, and increases intestinal permeability, thereby promoting a pro-inflammatory state that may exacerbate retinal pathology [28–30]. Similarly, diets deficient in dietary fiber and rich in ultra-processed foods diminish beneficial fiber-fermenting, short-chain fatty acid (SCFA)-producing species and promote the expansion of pro-inflammatory taxa, while compromising intestinal barrier function. This creates a systemic environment conducive to AMD pathogenesis [31, 32].

Furthermore, systemic comorbidities strongly associated with AMD risk—including hypertension, dyslipidemia, and diabetes—are themselves linked to distinct and often detrimental alterations in the gut microbiome [33–35]. These conditions share underlying pathways of chronic low-grade inflammation, endothelial dysfunction, and oxidative stress, all of which can be modulated by the gut microbiota and likely converge to affect retinal health. This interconnectedness underscores that interventions targeting gut dysbiosis in AMD should be considered part of a holistic therapeutic strategy. Addressing modifiable risk factors, such as smoking cessation, adoption of a Mediterranean-style diet, and management of systemic comorbidities, can synergistically improve microbial balance and may potentially slow AMD progression.

This review seeks to synthesize the evidence that gut microbiota influences the initiation and progression of AMD via the regulation of systemic and ocular immunity and inflammation. It also examines the clinical prospects and challenges associated with microbiome-targeted therapeutic strategies.

## The composition and function of the gut microbiota

The human gut constitutes a complex, individualized, and dynamic ecosystem in a state of equilibrium. It harbors approximately 10^14^ microorganisms, encompassing over 1000 species, with their concentration increasing progressively from the stomach to the distal colon. Biologically, gut microbiota are classified into seven hierarchical levels: kingdom, phylum, class, order, family, genus, and species, with species being the most fundamental unit. Based on natural characteristics, the predominant bacterial phyla are primarily classified into six major groups: *Firmicutes, Bacteroidetes, Proteobacteria, Actinobacteria, Verrucomicrobia, and Fusobacteria* [36]. Among these, *Firmicutes* account for approximately 60–80%, followed by *Bacteroidetes* at 20–30%, while other phyla constitute relatively minor proportions. A healthy gut microbiota is crucial for maintaining host homeostasis by actively participating in development, digestion, metabolism, and immune defense. However, when disruptions occur in its composition, function, or regulatory dynamics, the host’s immune system may exceed its tolerance for the microbiota, triggering an inflammatory state. This can subsequently lead to systemic tissue damage and the pathogenesis of various diseases. Based on their relationship with the host, gut microbiota can be categorized into three primary groups: i) Beneficial bacteria, primarily comprising *Bifidobacterium, Lactobacillus*, and *Bacteroides*, which represent the dominant flora and play a vital role in maintaining normal physiological functions. ii) Commensal opportunistic pathogens, such as *Enterococcus and Enterobacter*, which are typically harmless under conditions of microbial equilibrium but can become invasive under specific circumstances. iii) Pathogenic bacteria, often transient populations like *Proteus* and *Staphylococcus aureus*, which rarely achieve long-term colonization within the gut [37]. Gut microbiota dysbiosis is associated not only with gastrointestinal disorders but also with a spectrum of extra-intestinal diseases, including those affecting the neurological, metabolic, cancer-immunological, and cardiovascular systems [38–42].

During embryonic development, the retina and optic nerve originate from the brain and later develop as components of the central nervous system [43]. Studies have indicated that neurodegenerative diseases, such as Alzheimer’s disease and Parkinson’s disease, are associated with gut microbiota, sharing common pathological mechanisms with retinal diseases, including enhanced inflammation, impaired blood-brain barrier function, vascular dysfunction, and metabolic abnormalities [44, 45]. Rowan et al. demonstrated that dietary patterns influence the pathology of AMD, a process linked to gut microbiota, thereby introducing the concept of a “Gut-Eye axis [23].” This hypothesis posits that diet, probiotics, or antibiotics can modulate the progression of retinal diseases by altering the gut microbiota [46]. The subsequent detection of microbial presence in the systemic circulation [47], liver [48], pancreas [49], and even the eye [50] has further supported the potential significant role of gut microbiota in ocular pathologies.

**FIGURE 1:**
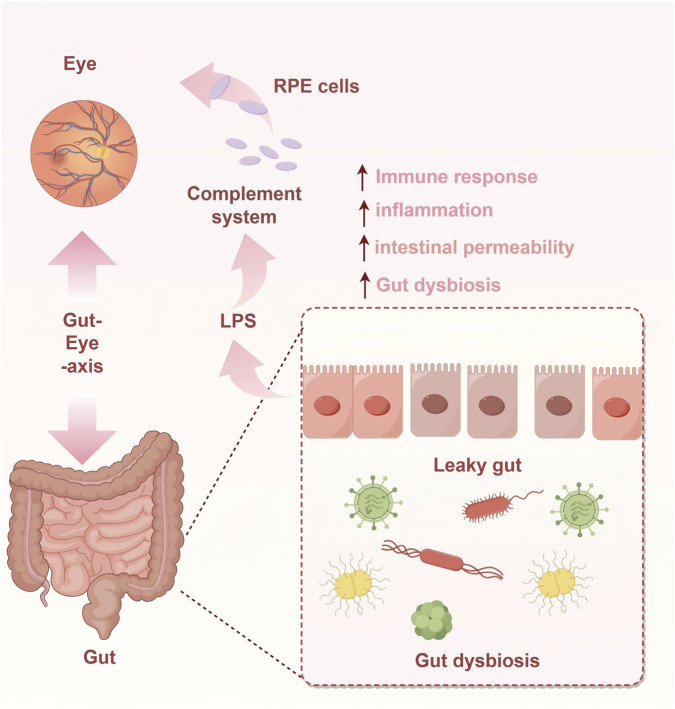
Mechanisms of Gut Microbiota in AMD.

Emerging evidence indicates that gut microbiota dysbiosis is associated with the pathogenesis of AMD [51, 52], suggesting that the Gut-Eye axis may represent a potential pathological origin of the disease. Mechanisms of Gut Microbiota in AMD (see Figure 1).

## Mechanisms underlying the influence of gut microbiota on AMD

### Characteristics of gut microbiota composition in AMD patients

Significant differences exist in the taxonomic composition of the gut microbiota between individuals with AMD and healthy subjects [53]. Clinical studies utilizing techniques such as 16S rRNA gene sequencing have revealed significant alterations in the gut microbiota of patients with AMD compared to healthy controls. A prominent finding is an increased *Firmicutes/Bacteroidetes* (F/B) ratio, which is considered a hallmark of gut dysbiosis [54]. The rise in *Firmicutes* abundance may contribute to elevated systemic inflammation in AMD patients, whereas the decrease in *Bacteroidetes* could compromise intestinal barrier integrity and disrupt nutrient metabolism [55]. Through metagenomic sequencing, Xue et al. identified additional features of gut microbial dysbiosis in AMD, including reduced alpha diversity, a decreased F/B ratio, and impaired activity in three degradation pathways: N-acetylneuraminate degradation, glycerol degradation to butanol, and glycogen degradation I. Notably, this study also provided the first evidence linking AMD to intestinal phage dysbiosis, identifying *Bacteroidaceae* as the primary host for AMD-associated phages. Collectively, these microbial alterations may promote increased intestinal permeability and bacterial translocation, thereby accelerating the formation and progression of vitreous deposits and RPE abnormalities [56]. Li et al. conducted gut microbiota and fecal metabolomic analyses in a laser-induced CNV mouse model alongside normal controls. Their findings revealed significant alterations in the gut microbiota of CNV mice, characterized by a marked upregulation of *Candidatus Saccharimonas* and relatively lower abundances of the *Prevotellaceae_NK3B31_group*, *Candidatus Soleaferrea*, and *Truepera*. Fecal metabolomics identified 73 altered metabolites. Further analysis demonstrated a significant correlation between the *Prevotellaceae_NK3B31_group* and *Candidatus Saccharimonas* [57]. The differences in gut microbiome composition between patients with AMD and healthy controls are summarized in Table 2.

**TABLE 2:** Gut microbiome differences between AMD patients and healthy controls.

Aspect of comparison	Patients with AMD	Healthy controls
Overall microbiome structure	Significant differences in microbial species and metabolic pathway abundance. Principal component analysis (PCA) reveals distinct clustering separate from controls	Relatively stable and distinct microbial community structure
Phylum/Class level differences	Higher relative abundance of Firmicutes	Higher relative abundance of Bacteroidetes
Genus/Species level differences (relative enrichment)	Anaerotruncus (associated with aging and inflammation) Oscillibacter (associated with high-fat diet) Ruminococcus torques (mucin-degrading capacity) Eubacterium ventriosum (linked to elevated pro-inflammatory cytokines) Prevotella (enriched in patients with advanced AMD)	Bacteroides eggerthii Other Bacteroides species
Core metabolic pathway differences	Enriched Pathways: • L-alanine fermentation • Glutamate degradation • Arginine biosynthesis • Purine ribonucleoside degradation	Enriched Pathways: • Fatty acid elongation pathway
Potential mechanistic links	• Increased gut permeability • Immune & complement system • Nutrition & metabolism	A homeostatic gut microbiome

### Gut microbiota metabolites and AMD

Gut microbiota-derived metabolites are highly diverse and play a significant role in the pathogenesis of AMD [58]. SCFAs, including acetate, propionate, and butyrate, are major products of dietary fiber fermentation by gut microbes. SCFAs regulate host metabolism and immune function through multiple pathways [59, 60]. In AMD, abnormal SCFA levels may disrupt retinal cell metabolism and inflammatory responses. For example, butyrate exerts anti-inflammatory effects by inhibiting the NF-κB signaling pathway and reducing the production of inflammatory cytokines. A reduction in butyrate-producing bacteria in AMD patients leads to decreased butyrate levels, which may attenuate its anti-inflammatory protection in the retina and thereby promote AMD progression.

Further supporting the therapeutic relevance of this axis, Zhang et al [61] demonstrated that metformin may exert protective effects in neovascular AMD by modulating the gut microbiota and the Gut-Eye axis. Specifically, metformin treatment significantly altered gut microbial composition, increasing the abundance of *Bifidobacterium* and *Akkermansia*, and elevated fecal concentrations of butyrate, other SCFAs, and Bile acids. These metabolites are considered key mediators of its protective effects: butyrate inhibits pathological angiogenesis via the TXNIP/VEGFR2 signaling pathway, while SCFAs activate the G-protein coupled receptors GPR41 and GPR43 on intestinal epithelial cells, subsequently triggering the MAPK pathway and promoting the secretion of chemokines and cytokines, which may ultimately modulate retinal inflammation and angiogenesis [62].

In addition, gut microbiota metabolize tryptophan into indole derivatives, such as indole-3-lactic acid and indole-3-propionic acid (IPA). These metabolites can activate the aryl hydrocarbon receptor (AHR), thereby regulating immune cell function and retinal tissue homeostasis, and influencing the development of ocular diseases [63].

### Intestinal barrier integrity and AMD

The intestinal mucosal epithelium forms a physical barrier through tight junction proteins, such as occludin and claudin family proteins, which prevents gut bacteria and their metabolites from entering the systemic circulation. However, damage to this barrier—caused by factors such as gut dysbiosis, dietary insults, or oxidative stress—can lead to a “leaky gut” condition, resulting in increased intestinal permeability. Under conditions of “leaky gut,” bacteria and their metabolites, such as lipopolysaccharide (LPS), can translocate across the compromised intestinal mucosal barrier into the systemic circulation. As a key inflammatory trigger, LPS specifically binds to Toll-like receptor 4 (TLR4) expressed on immune cells—including monocytes and macrophages. This binding activates intracellular inflammatory signaling pathways, such as NF-κB, leading to the robust secretion of pro-inflammatory cytokines by these immune cells [64]. Among these cytokines, IL-6 amplifies the inflammatory cascade by activating the JAK-STAT signaling pathway and promotes vascular endothelial cell activation [65]. Meanwhile, TNF-α directly induces apoptosis and enhances the expression of other inflammatory mediators, such as the chemokine CCL2, thereby further recruiting inflammatory cells to the site [66].

These LPS-induced pro-inflammatory cytokines serve as a critical link between “leaky gut” and AMD. A central pathological feature of AMD is dysfunction of the RPE, a cell layer essential for maintaining retinal homeostasis through its barrier and phagocytic functions. When cytokines such as IL-6 and TNF-α reach the retina via systemic circulation, they contribute to AMD pathogenesis through multiple mechanisms [67]. These cytokines contribute to AMD pathogenesis through several key mechanisms. Notably, they can disrupt tight junctions between RPE cells, which exacerbates damage to the blood-retinal barrier and impairs the phagocytic capacity of RPE cells. This dysfunction leads to the accumulation of metabolic waste, thereby promoting the formation of drusen—a hallmark of Dry AMD.

A well-defined molecular link exists between IL-6 and CNV in Wet AMD. IL-6 binding to its receptor complex (IL-6R/gp130) on retinal cells activates the JAK/STAT3 signaling pathway. Following phosphorylation and dimerization, STAT3 translocates to the nucleus and directly binds specific response elements in the VEGF gene promoter, driving its transcription [68]. IL-6 can additionally activate the Ras/MAPK pathway, further amplifying VEGF expression. The consequent elevation in VEGF levels induces abnormal proliferation of choroidal capillary endothelial cells, facilitating their penetration through the compromised Bruch’s membrane into the subretinal space to form CNV [69]. These fragile neovessels are prone to leakage and hemorrhage, leading to acute vision loss—a defining characteristic of Wet AMD.

Concurrently, TNF-α exacerbates oxidative stress and drives RPE degeneration through multifaceted mechanisms. Upon binding to TNFR1 on RPE cells, TNF-α activates the NF-κB pathway, upregulating NADPH oxidases such as NOX4 and increasing intracellular reactive oxygen species (ROS) [70]. It also impairs mitochondrial electron transport chain function, promoting mitochondrial ROS production. The resulting ROS overload damages cellular proteins, lipids, and DNA, disrupting homeostasis [71]. Furthermore, TNF-α activates the p38 MAPK pathway, upregulating senescence-associated proteins including p53 to induce RPE cell senescence, and initiates the apoptotic cascade via caspase-8, leading to caspase-3-dependent apoptosis [72, 73]. Collectively, these TNF-α-mediated processes contribute to RPE cell loss, compromise the outer blood-retinal barrier, and accelerate the progression to advanced AMD features such as drusen expansion and GA [74].

### Gut microbiota and the complement system

The complement system, a crucial component of innate immunity, can be initiated through three pathways: the lectin, classical, and alternative pathways. Accumulating evidence indicates a close relationship between the gut microbiota and the complement system. For example, Wu et al. reported that the production of complement component 3 (C3) in the host intestine is associated with gut microbial abundance, and its baseline level is influenced by microbial composition in both humans and mice, exhibiting inter-individual variation [75]. Complement activation is also an important factor influencing the progression of AMD. Specifically, complement factor H (CFH), a key regulator of complement activation, is strongly linked to the genetic risk of AMD. The CFH Y402H polymorphism has been associated with alterations in various microbial taxa and leads to elevated levels of the membrane attack complex in carriers, which may exacerbate inflammatory responses and thereby promote AMD development [76]. In a long-term follow-up cohort of patients with intermediate AMD, Anne M. et al. identified significant associations between progression to late AMD and several systemic complement factors and ratios—such as C4, C4b, C3a/C3, C5a/C5, sC5b-9/C5, and factor I—with some indicators showing hazard ratios exceeding tenfold [77]. In 2023, the FDA approved two intravitreal injectable complement inhibitors: pegcetacoplan (SYFOVRE), which targets C3, and avacincaptad pegol (IZERVAY), which targets C5. This milestone marks the entry of AMD treatment into the “complement inhibition era” and provides the first disease-modifying therapy for patients with GA [78, 79].

Furthermore, research by Denise C. Zysset-Burri et al. revealed consistent alterations in both human AMD patients and C3-deficient mice, including enrichment of Firmicutes, reduction in *Bacteroidetes*, and dysregulation of purine metabolism pathways. These findings suggest that the complement system may indirectly influence AMD progression by modulating the composition and function of the gut microbiome. Specifically, the class *Negativicutes* showed a positive correlation with CFH and may exacerbate AMD via alternative pathway activation, whereas the genus *Bacteroides* likely confers protection by suppressing excessive complement activation [80]. These results align with the same team’s earlier findings from 2017 and provide additional evidence linking the complement system to AMD pathogenesis, further supporting the notion that the gut microbiome and complement system are interconnected and may jointly contribute to AMD development [54].

### Investigating the contributions of gut microbiota to AMD pathogenesis and modulation in animal models

Aimée Parker et al. employed an integrated approach combining metagenomic sequencing, NMR-based metabolomics, and immunohistochemistry to investigate the role of gut microbiota in regulating age-related damage to the intestinal barrier, Central Nervous System, and retina. Their findings demonstrated that fecal microbiota transplantation (FMT) from aged donors to young recipients significantly accelerated intestinal barrier leakage, activated microglia in the brain, and induced retinal inflammation, evidenced by elevated complement C3 and reduced RPE65 protein levels. Conversely, when aged mice received microbiota from young donors, complement C3 and RPE65 levels were restored, underscoring the critical role of gut microbiota in modulating inflammatory processes relevant to AMD [81].

Andriessen et al. reported that mice fed a high-fat diet developed gut dysbiosis characterized by an elevated F/B ratio and increased intestinal permeability. These animals exhibited a chronic low-grade inflammatory state, marked by elevated levels of TNF-α, IL-6, IL-1β, and VEGF-A. Such inflammatory mediators are known to exacerbate CNV, thereby promoting the development of neovascular AMD. Furthermore, studies have shown that increased abundances of *Anaerotruncus* and *Ruminococcaceae* in aged mice correlate with elevated serum levels of MCP-1. As a member of the chemokine family, MCP-1 recruits monocytes/macrophages into the retina, stimulating the secretion of TNF-α, IL-1β, and VEGF, and thereby contributing to retinal inflammation and angiogenesis. These findings suggest that gut microbiota may modulate inflammatory responses via mediators such as TNF-α and MCP-1, thereby influencing the pathogenesis of AMD [82]. The key mechanisms linking gut microbiota dysbiosis to the pathogenesis of AMD are summarized in Table 3.

**TABLE 3:** Summary of key mechanisms linking gut microbiota to AMD pathogenesis.

Mechanistic pathway	Key alterations/Components	Proposed impact on Retina/AMD
Microbial composition & diversity	↑ F/B ratio; ↓ alpha diversity; alterations in specific genera such as candidatus saccharimonas	Promotes a pro-inflammatory systemic state; compromises gut barrier integrity
Microbial metabolites	↓ SCFAs including butyrate; altered tryptophan derivatives such as indole-3-propionic acid	Reduces anti-inflammatory and anti-angiogenic protection; disrupts immune homeostasis via AhR signaling
Intestinal barrier integrity (“leaky gut”)	Impaired tight junctions; ↑ intestinal permeability; systemic translocation of LPS	Triggers chronic low-grade inflammation; elevates circulating pro-inflammatory cytokines such as IL-6 and TNF-α
Systemic & ocular inflammation	LPS/TLR4/NF-κB activation; ↑ IL-6, TNF-α, MCP-1	IL-6 drives VEGF-mediated CNV; TNF-α induces RPE oxidative stress, senescence, and apoptosis; recruits immune cells to retina
Gut-complement system Interaction	CFH polymorphism-linked microbial shifts; complement activation involving C3, C5a, and MAC	Exacerbates inflammatory response; synergistically damages RPE and choroid

## Therapeutic strategies targeting the gut microbiome

Given the established links between gut dysbiosis and AMD pathogenesis—particularly through mechanisms involving microbial metabolites, intestinal barrier integrity, and systemic/complement inflammation—targeted modulation of the gut microbiome emerges as a promising therapeutic avenue. Current strategies aim to restore microbial equilibrium, enhance beneficial metabolite production, and mitigate pro-inflammatory cascades, thereby potentially influencing the course of AMD.

### Dietary interventions

Diet is a central driver in shaping the gut microbiota, influencing the host metabolome through microbial metabolism. These diet and microbiota-related metabolites subsequently affect the initiation and progression of AMD via the Gut-Eye axis. Rowan et al. demonstrated that an HG diet induced multiple AMD-like features in mice, including RPE atrophy, lipofuscin accumulation, and photoreceptor degeneration. In contrast, an LG diet prevented such pathological changes. Even when initiated in aged mice, switching from an HG to an LG diet halted or reversed AMD characteristics. Mechanistically, the LG diet reduced the accumulation of advanced glycation end products (AGEs) and lipid peroxidation products such as CEP and 4-HNE. It also modulated the gut microbiota—with *Clostridiales* associated with HG/AMD susceptibility and *Bacteroidales* linked to LG/AMD protection—and increased levels of protective microbial metabolites such as serotonin. These effects collectively underlie the protective role of the LG diet against diet- and age-induced AMD, mediated through the Gut-Eye axis [23]. Studies have shown that compared to the Western diet rich in sugar and fat, the Mediterranean diet—characterized by high consumption of fish, vegetables, olive oil, and moderate intake of meat—is associated with a reduced risk of progression to late-stage AMD, Fish and vegetables are identified as key protective components of this dietary pattern [83, 84]. Its protective effects are mediated through several gut-centric mechanisms: (1) It promotes a higher abundance of Bacteroidetes and a lower Firmicutes/Bacteroidetes ratio, a profile linked to lower systemic inflammation. (2) It enhances the production of SCFAs like butyrate, which strengthen the intestinal epithelial barrier and exert systemic anti-inflammatory effects. (3) By improving barrier function and reducing systemic inflammatory tone such as lowering TNF-α, IL-6, it indirectly dampens microglial activation in the retina and the propensity for CNV [51]. Thus, the Mediterranean diet acts as a multi-modal intervention targeting metabolite production, barrier integrity, and inflammatory pathways.

### Pharmacological and Biological Agents

Certain drugs or natural metabolites can exert part of their therapeutic effects through gut microbiota remodeling, thereby linking pharmaceutical intervention to the Gut-Eye axis. Metformin, beyond its glucose-lowering effects, has shown promise in neovascular AMD models. Zhang et al. demonstrated that its protective effect is mediated via gut microbiota modulation [61]. Specifically, metformin treatment increases the abundance of beneficial genera including *Bifidobacterium* and *Akkermansia*, and elevates fecal levels of SCFAs and Bile acids. The subsequent increase in butyrate inhibits pathological angiogenesis through the TXNIP/VEGFR2 pathway, providing a direct example of how a drug can harness the SCFA-mediated mechanism to achieve ocular therapeutic effects. Prasad’s research confirmed that direct supplementation of IPA and other related interventions improve glucose homeostasis and retinal function. This is achieved by regulating the gut microbiota, enhancing intestinal barrier function, activating the AhR and pregnane X receptor (PXR), and inhibiting the TLR4/NLRP3 inflammatory pathway [85].

Additionally, the recent FDA approval of complement C3 and C5 inhibitors (pegcetacoplan, avacincaptad pegol) for GA represents a breakthrough in AMD treatment [78, 79]. The complement system and gut microbiome are known to be interrelated: studies have shown that complement factor polymorphisms such as CFH Y402H are associated with distinct gut microbial profiles [76], and complement deficiency such as C3 deficiency alters gut microbiota in mice [80]. Although current complement inhibitors are administered intravitreally, their systemic effects or the status of the gut-complement axis may influence treatment response. Therefore, future research into oral agents that modulate the complement system, such as those acting via factor I, should consider their impact on the gut microbiome or potential synergistic effects with the gut microbiome as part of their mechanism of action.

### Probiotics and prebiotics

Probiotics are recognized for their ability to inhibit pathogen colonization, modulate gut microbiota and immune responses, and enhance intestinal epithelial barrier function [86]. By primarily acting through the Gut-Eye axis, orally administered probiotics represent a systemic therapeutic strategy with potential relevance to retinal diseases like AMD. In a randomized controlled trial involving 57 AMD patients, an 8-week oral probiotic intervention containing *Bacillus*, *Lactobacillus*, and *Bifidobacterium* strains did not lead to significant improvements in clinical symptoms or other metabolic parameters. However, it exerted positive effects on systemic oxidative stress markers by reducing the pro-oxidant malondialdehyde (MDA) and enhancing total antioxidant capacity (TAC) [87]. This mechanistic link is relevant because oxidative stress contributes to the pathology of both Dry and Wet AMD. By reducing systemic oxidative load, probiotics may help lower the risk of damage to the RPE and photoreceptors.

Studies in animal models of retinal degeneration have shown that specific probiotic strains can attenuate photoreceptor cell death, reduce retinal glial activation, and improve visual function, likely through the modulation of systemic and local immune responses [88, 89]. While direct translation to human AMD requires validation, these findings underscore the potential of probiotics not merely as gut modulators, but as agents capable of influencing the neuroinflammatory milieu of the retina itself. Future research should focus on identifying strain-specific effects, optimal dosing regimens, and their potential role as adjunctive therapy to standard AMD treatments.

Prebiotics are substrates selectively utilized by host gut microorganisms, conferring health benefits to the host [90, 91]. A defining characteristic of prebiotics is their resistance to degradation by host enzymes [91]. By selectively stimulating the growth and activity of beneficial bacteria such as *Lactobacillus* and *Bifidobacterium*, prebiotics modulate the composition of the gut microbiota and thereby enhance the functional efficacy of probiotics [92].

In the context of ocular diseases, a double-blind, randomized controlled trial investigated the potential of oral probiotics and prebiotics in managing dry eye disease. The results demonstrated that after 4 months of intervention, the mean Ocular Surface Disease Index score in the treatment group was significantly improved compared to the control group [93]. Nevertheless, research on prebiotics in ocular pathologies remains limited, and their specific contributions to the observed effects require further clarification.

### FMT

FMT involves transferring functional microbial communities from the feces of healthy donors into the gastrointestinal tract of recipients to reconstitute a balanced gut microbiota. This procedure enhances microbial diversity, restores Bile acids metabolism, and improves intestinal function, demonstrating notable efficacy in eradicating *Clostridium difficile* infection in patients with colitis [94]. In adults, the gut microbiota typically maintains relative stability; however, its diversity gradually declines with aging. Compared to younger individuals, older persons subjects exhibit significant alterations in microbial composition, characterized by reduced abundances of *Bacteroides*, *Bifidobacterium*, and *Enterobacteriaceae*, alongside a relative increase in *Clostridia* [95].

However, research on the application of FMT for treating AMD remains limited. In the context of ocular disorders, Jiao et al. demonstrated that aging induces gut microbial dysbiosis in mice, which in turn triggers chronic inflammation, lipid accumulation, and circadian disruption in the lacrimal gland. Importantly, FMT from young donors reversed these pathological changes by remodeling the gut microbiota, thereby restoring lacrimal secretory function and circadian transcriptional rhythms [87]. Aimée Parker et al. demonstrated that FMT from aged donors to young recipients significantly accelerated intestinal barrier leakage, activated cerebral microglia, and induced retinal inflammation, characterized by elevated complement C3 and reduced RPE65 levels. Conversely, aged mice receiving young donor microbiota showed reversal of these biomarkers, with normalized complement C3 and RPE65 expression. These findings suggest that FMT may represent a potential therapeutic strategy for AMD [81]. Table 4 provides a comparative overview of these microbiome-targeted therapeutic strategies.

**TABLE 4:** Overview of microbiome-targeted therapeutic strategies for AMD.

Strategy	Approach	Proposed mechanisms of action in AMD context	Current evidence & considerations
Dietary interventions	Mediterranean diet; low-glycemic diet	Modulates microbiota composition (↑Bacteroidetes, ↓F/B ratio); ↑SCFAs production; strengthens gut barrier; reduces systemic inflammation	Strong epidemiological association with reduced AMD risk; causal evidence from animal models
Pharmacological agents	Metformin; IPA	Remodels gut microbiota (↑beneficial genera); increases butyrate and IPA; activates AhR/PXR; inhibits TLR4/NLRP3 inflammation	Preclinical evidence promising; human studies needed to confirm efficacy for AMD
Probiotics	Oral supplements containing strains such as *Lactobacillus* and bifidobacterium	Competes with pathogens; modulates host immunity; enhances gut barrier; may reduce systemic oxidative stress	Preclinical evidence promising; human studies needed to confirm efficacy for AMD
Prebiotics	Dietary fibers including inulin and FOS, which selectively feed beneficial bacteria	Stimulates growth of beneficial bacteria such as *Lactobacillus*, bifidobacterium; synergizes with probiotics (synbiotics)	Limited direct research in AMD; shown benefit in related ocular surface diseases
FMT	Transfer of processed fecal matter from a healthy donor	Most comprehensive restoration of microbial diversity and function; repairs gut barrier; reverses pro-inflammatory state	Preclinical studies show reversal of AMD-related biomarkers; clinical application in AMD remains exploratory

## Discussion

Advances in microbiomics have established clear connections between gut microbiota and systemic diseases. The concept of the “Gut-Eye axis” offers a new lens through which to understand the pathogenesis of ocular conditions such as AMD. Evidence indicates that gut dysbiosis influences the onset and progression of AMD through multiple pathways, including microbial metabolites, immune regulation, barrier integrity, and the complement system [96, 97]. This perspective not only highlights the significant role of intestinal microecology in ophthalmology but also provides a theoretical foundation for clinical intervention.

Studies reveal significant differences in gut microbiota characteristics between AMD patients and healthy individuals. These primarily manifest as an altered Firmicutes/Bacteroidetes ratio, reduced diversity, and shifts in the abundance of specific bacterial genera [98]. These structural changes are closely linked to functional disturbances. Reduced levels of SCFAs diminish their anti-inflammatory effects, while abnormalities in tryptophan metabolism disrupt immune homeostasis. More critically, gut dysbiosis can compromise intestinal barrier function, allowing pro-inflammatory molecules like LPS to enter the systemic circulation and trigger a state of chronic, low-grade inflammation. This inflammatory environment, driven by factors such as IL-6 and TNF-α, directly or indirectly damages retinal pigment epithelial function, exacerbates oxidative stress, and promotes the formation of CNV.

Animal experiments provide further evidence for a causal relationship for the existence of the Gut-Eye axis. Diet-induced dysbiosis can accelerate AMD-like pathology [82]. FMT studies demonstrate that microbiota from aged donors is sufficient to induce retinal inflammation in younger recipients, while microbiota from young donors can reverse retinal abnormalities in aged subjects [81].

The Gut-Eye axis theory suggests that strategies targeting the gut microbiome hold substantial clinical value. For prevention, personalized dietary adjustments or specific prebiotic interventions to optimize microbial balance may serve as low-risk strategies to delay disease progression. Therapeutically, probiotic formulations have already shown potential in improving oxidative stress in AMD patients and could become a valuable adjunct to existing treatments. Furthermore, the individualized nature of gut microbiota introduces a new dimension for precision medicine. Analyzing microbial profiles could enable patient stratification and the development of tailored intervention protocols.

However, challenges remain in this field. The precise causal mechanisms linking microbiota and AMD require further clarification. Standardized protocols for probiotic applications are yet to be established, and the use of FMT in ophthalmology demands rigorous evaluation. Future research should focus on elucidating key mechanisms, conducting well-designed clinical trials, and developing personalized treatment frameworks based on multi-omics data. Integrating gut microbiome modulation into ophthalmic practice represents a promising step toward precision medicine in eye care.
